# The Effect of Colistin Resistance-Associated Mutations on the Fitness of *Acinetobacter baumannii*

**DOI:** 10.3389/fmicb.2016.01715

**Published:** 2016-11-01

**Authors:** Xinli Mu, Nanfei Wang, Xi Li, Keren Shi, Zhihui Zhou, Yunsong Yu, Xiaoting Hua

**Affiliations:** ^1^Department of Infectious Diseases, Sir Run Run Shaw Hospital, College of Medicine, Zhejiang UniversityHangzhou, China; ^2^State Key Laboratory for Diagnosis and Treatment of Infectious Disease, First Affiliated Hospital, College of Medicine, Zhejiang UniversityHangzhou, China

**Keywords:** *Acinetobacter baumannii*, colistin, resistance mechanism, fitness cost, whole genome sequencing

## Abstract

*Acinetobacter baumannii* had emerged as an important nosocomial and opportunistic pathogen worldwide. To assess the evolution of colistin resistance in *A. baumannii* and its effect on bacterial fitness, we exposed five independent colonies of *A. baumannii* ATCC 17978 to increasing concentrations of colistin in agar (4/5) and liquid media (1/5). Stable resistant isolates were analyzed using whole genome sequencing. All strains were colistin resistant after exposure to colistin. In addition to the previously reported *lpxCAD* and *pmrAB* mutations, we identified four novel putative colistin resistance genes: *A1S_1983. hepA. A1S_3026*, and *rsfS*. Lipopolysaccharide (LPS) loss mutants exhibited higher fitness costs than those of the *pmrB* mutant in nutrient-rich medium. The colistin-resistant mutants had a higher inhibition ratio in the serum growth experiment than that of the wild type strain in 100% serum. Minimum inhibitory concentration (MIC) results showed that the LPS-deficient but not the *pmrB* mutant had an altered antibiotic resistance profile. The compensatory mutations partially or completely rescued the LPS-deficient’s fitness, suggesting that compensatory mutations play an important role in the emergence and spread of colistin resistance in *A. baumannii*.

## Introduction

*Acinetobacter baumannii* had emerged as an important nosocomial and opportunistic pathogen worldwide, especially in intensive care units (Peleg et al., 2008). *A. baumannii* treatment is difficult because the clinical strains generally show resistance to multiple antibiotics (Peleg et al., 2008). Because the extensively drug-resistant isolates demonstrated sensitivity only to colistin, colistin has become the only clinically useful agent against these pathogens (Falagas et al., 2005; Giske et al., 2008; Magiorakos et al., 2012). However, due to its low efficacy, colistin should be used in combination antibiotic therapy for the treatment of XDR *A. baumannii* (Durante-Mangoni et al., 2014).

Colistin is a polycationic antimicrobial peptide that targets the polyanionic bacterial lipopolysaccharide (LPS) of Gram-negative bacteria. There are two different colistin resistance mechanisms (Beceiro et al., 2014). The first involves the inactivation of the lipid A biosynthesis pathway and the complete loss of surface LPS. This loss might be caused by mutations in *lpxC. lpxA*, or *lpxD*. Isolates harboring these mutations showed high colistin minimum inhibitory concentrations (MICs), although their fitness cost was also high (Beceiro et al., 2014; Wand et al., 2015). The second resistance mechanism is mediated by the *pmrAB* two-component system. Mutations in *pmrA* and *pmrB* induce the activation of *pmrC*, which adds phosphoethanolamine (PEtn) to the hepta-acylated form of lipd A (Beceiro et al., 2011). The *pmrB* mutation did not result in growth retardation (Durante-Mangoni et al., 2015; Wand et al., 2015).

Antimicrobial pressure drives the evolution of antimicrobial resistance in bacteria (Baker et al., 2013), and resistance is often associated with reduced bacterial fitness. Epistasis can compensate for the fitness cost of these mutants (Andersson and Hughes, 2010). Epistasis occurs not only within the same resistance pathway but also in different resistance pathways. In fluoroquinolone resistance, the *parC* mutation had the greatest effect on increase fitness of low fitness strains that harbored mutations in *gyrA* and *marR* (Marcusson et al., 2009). Moreover, the cost of multiple resistance was smaller than expected during the evolution of multi-drug resistance (Trindade et al., 2009). Understanding the fitness cost of the evolution of resistance is important to control the spread of resistant bacteria (Baker et al., 2013).

To assess the evolution of *A. baumannii* colistin resistance, we evolved *A. baumannii* through continuous exposure to colistin in agar plates and liquid media. Four novel putative colistin resistance genes were identified in addition to *lpxCAD* and *pmrAB*. Fitness and resistance compensatory mutations appeared in the bacteria exposed to colistin.

## Materials and Methods

### Bacterial Isolates and Antimicrobial Susceptibility Testing

All bacteria used in this study were the ATCC 17978 strain and its laboratory-evolved mutants of *A. baumannii*. The MICs were determined by broth dilution using Etest (AB Biodisk, Solna, Sweden) on Mueller-Hinton (MH) agar. The results were interpreted according to the CLSI or EUCAST breakpoints (Clinical and Laboratory Standards Institute, 2014; European Committee on Antimicrobial Susceptibility Testing, 2015). The bacteria were cultured in Luria–Bertani (LB) medium at 37°C.

### Laboratory-Evolved Colistin Resistant Mutants

Four independent single colonies of *A. baumannii* ATCC 17978 were grown overnight at 37°C. The *in vitro* serial passage experiments were performed using three methods. First, the bacteria were streaked on LB agar plates containing 10 mg/L colistin once each day for 15 days and then incubated at 37°C. Then, the bacteria were serially passaged for another 15 days in the presence of 50, 100, and 200 mg/L colistin. Second, the bacteria were streaked on LB agar plates containing 10 mg/L colistin once each day for 60 days and then incubated at 37°C. Third, the cultures were exposed to serially increasing concentrations of colistin, starting from 1/2 the MIC and doubling every day for 12 days. The overnight cultures were stored at -80°C prior to the experiments and analysis.

### Whole Genome DNA Sequencing and Analysis

Bacteria from a single colony were cultured overnight at 37°C in MH broth. The genomic DNA was extracted using a QIAamp DNA minikit (Qiagen, Valencia, CA, USA) following the protocol of the manufacturer (Hua et al., 2012). Agarose gel electrophoresis and a NanoDrop spectrophotometer (ND-1000, ThermoFisher, Waltham, MA, USA) were used to determine the quality and quantity of the extracted genomic DNA. The 300 bp library used for Illumina paired-end sequencing was constructed from 5 μg of genomic DNA purified from the laboratory-evolved mutants. Mapping and SNP detection were performed using breseq (Deatherage and Barrick, 2014). The regions containing the detected SNPs were amplified by PCR. The PCR products were sent to Biosune (Biosune, Hangzhou, China) for Sanger sequencing. Duplications were detected using the CLC genomic workbench (8.02.2) and were verified by real-time PCR (**Supplementary Table S2**).

### Fitness Cost and Serum Resistance Measurement

Four independent cultures per strain were grown overnight in MH (Mueller–Hinton), diluted 1:1000 [MH or fetal bovine serum (FBS)] and aliquoted into a flat-bottom 96-well plate in four replicates. The plate was agitated at 37°C. The OD_600_ of each culture was determined every 5 min for 16 h using a the BioTEK Synergy plate reader (Biotek, Winooski, VT, USA). The growth rate was estimated by an R script based on the OD_600_ curves (Li et al., 2015). Fitness data are shown in the **Supplementary Table S1**. Statistical analyses were performed using R 3.2.1 (R Development Core Team, 2015). Two-way analysis of variance (ANOVA) and TukeyHSD analysis were used to assess differences between the means, with a significant probability at a *P* value of ≤0.05.

## Results

### Mutations in Laboratory-Evolved Colistin Resistant Strains

To investigate the colistin resistance mechanism in *A. baumannii*, we evolved *A. baumannii* ATCC 17978 in agar plates and liquid media with increasing or constant concentrations of colistin. A strain description and the MICs for colistin of the laboratory-evolved strains are listed in **Supplementary Table S1**. The MIC of ATCC 17978 for colistin was 0.19 mg/L. The MICs of the laboratory-evolved strains ranged from 6 mg/L to > 256 mg/L. The final isolates from each colony were sent for whole genome sequencing (WGS) to identify mutations. The mutations in the laboratory-evolved strains were detected via breseq, which mapped the sequence reads onto the ATCC 17978 reference genome. Then, the generation time each mutation appeared during the experiment was determined by PCR and sanger sequencing.

**FIGURE 1:**
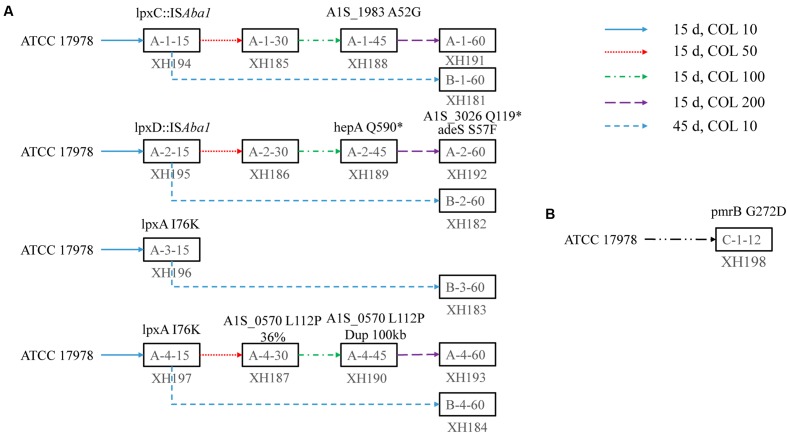
**The evolutionary pathway of colistin resistance in *Acinetobacter baumannii* 17978 in solid agar and liquid medium.** **(A)** Four colonies of *A. baumannii* ATCC 17978 were inoculated in a solid agar plate containing 10 mg/L colistin and serially passaged for 15 days. Then, one colony was continuously cultured with 10 mg/L colistin on agar plates for 45 days. Another colony was streaked on 50 mg/L colistin plates for a 15 days serial passage. Then, the bacteria were streaked onto 100 and 200 mg/L colistin plates for 15 days. The strains were stored at -80°C every 15 days. The final strains were sent for whole genome sequencing. The mutations were detected by breseq, which mapped the sequenced reads onto the reference genome. The mutation sites in the final strain were identified by PCR and Sanger sequencing. The mutations related to colistin resistance or fitness are labeled above the strain. **(B)** One lineage of *A. baumannii* 17978 was inoculated in Mueller Broth (MH) medium with increasing concentrations of colistin for 12 days. The final strains were sent for whole genome sequencing. The mutations were detected by breseq, which mapped the sequenced reads onto the reference genome. The mutations related to colistin resistance or fitness are labeled above the strain.

The mutations that occurred in the early stage of evolution were found in *pmrB. lpxC. lpxA*, and *lpxD* (**Figure 1**). Additional mutations were detected in *A1S_1983. hepA* (A1S_2462), *A1S_3026. rsfS* (A1S_0570), and *adeS* (A1S_1754) (**Supplementary Tables S3** and **S4**).

### Colistin Resistance had a Fitness Cost

Among the mutations detected, *A1S_1983. hepA. A1S_3026*, and *rsfS* were hypothesized to be involved in colistin resistance. Conversely, the *adeS* mutation was not involved in colistin resistance (data not shown). The ratio of the *A1S*_0570 mutation in A-4-30 (XH187) was 36%, and this ratio increased to 100% in A-4-45 (XH190).

**FIGURE 2:**
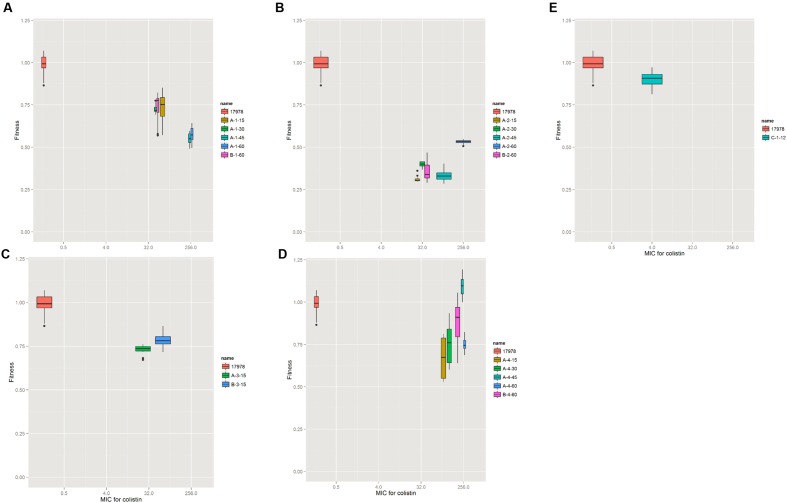
**Resistance and fitness of the wild type and evolved strains.** For the wild type and evolved strains, the relative fitness is shown as a function of the minimum inhibitory concentration (MIC) for colistin. Each box symbol represents one strain. The relative fitness was determined by measuring the growth rate in MH medium at OD600. The growth rate was calculated from the OD_600_ values during exponential growth by an R script. **(A–D)** Four colonies of *A. baumannii* strains evolved in solid agar plates. **(E)** The strain evolved in liquid media.

**FIGURE 3:**
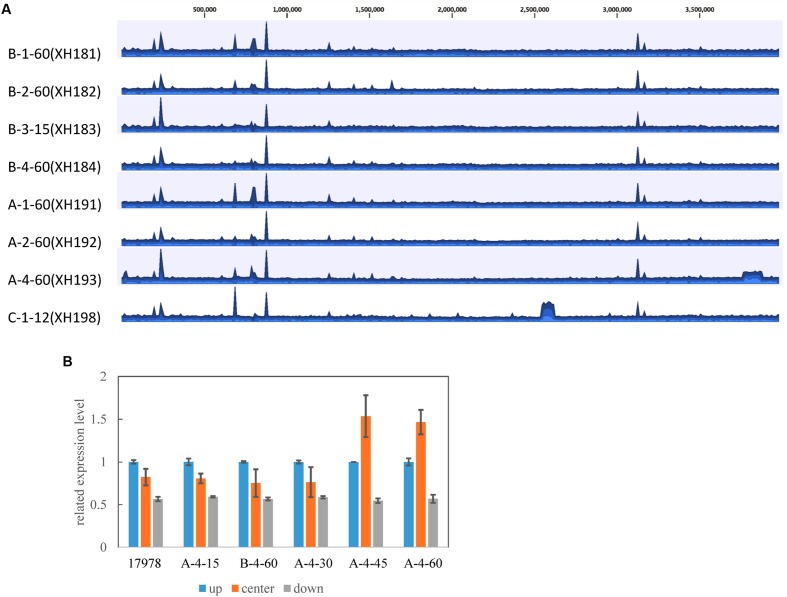
**(A)** Coverage of colistin-resistant strains derived from *A. baumannii* ATCC 17978. Each plot shows the coverage for the alignment of each strain to the ATCC 17978 reference strain. The mean coverage for each strain is denoted by a curve (blue). Duplicated genomic segments appear as regions showing two or more mean coverages. **(B)** Confirmation of the 100 kb duplication in the colistin-resistant strains by RT-PCR. The genomic DNA was obtained from the strains and used as templates for RT-PCR. Three RT-PCR primers were designed upstream (up, A1S_3272), in the center (A1S_3302), and downstream (down, A1S_3366) of the duplication fragment. The error bars represent the SE from three replicates.

To investigate the effect of the mutations on *A. baumannii* fitness, we measured the growth rate of the laboratory-evolved strains as a proxy for fitness. The strains selected in the agar plates showed a high fitness cost in the first 15 selected generations, while increased resistance and fitness compensation were observed later in the later selection process (**Figure 2**). The evolution in colistin resistance and fitness was diverse among the four lineages. In lineage 1, no fitness compensation was observed while resistance to colistin was increased (**Figure 2A**). In lineage 2, the fitness was partially recovered with a mutation in *A1S_3026*, and the resistance to colistin increased (**Figure 2B**). Due to contamination, lineage 3 did not have enough isolates for analysis. In lineage 4, the fitness was completely recovered with a mutation in *A1S_0570*, while the resistance increased (**Figure 2D**). A 100 kb duplication occurred in A-4-45(XH193) and A-4-60(XH190; **Figure 3**), which showed a higher fitness compared to that of A-4-30(XH187). These results indicated that the duplication of the long chromosome fragment may play a role in the bacterial fitness. In addition, the growth of strains harboring the *pmrB* mutation was slightly retarded (**Figure 2E**).

### Lipopolysaccharide Affects Serum Resistance

**FIGURE 4:**
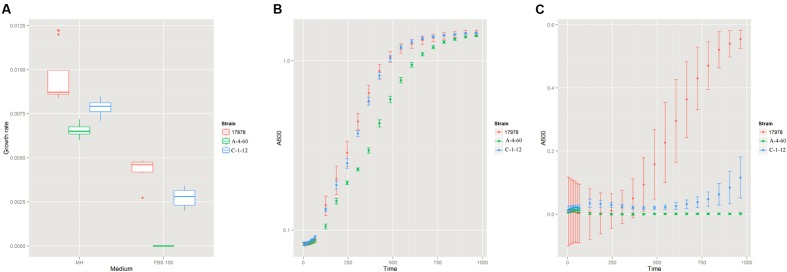
**(A)** The growth rate of the wild type strain (ATCC 17978), the LPS (Lipopolysaccharide)-deficient mutant (A-4-60, XH193) and the LPS-modified mutant (C-1-12, XH198) in MH and fetal bovine serum (FBS). The wild type strain (ATCC 17978), LPS-deficient mutant (A-4-60, XH193), and LPS-modified mutant (C-1-12, XH198) were grown in MH and FBS at 37°C. The growth rates of the strains were determined by measuring the OD_600_ every 5 min and were estimated by an R script based on the OD_600_ curves. Each strain represents four biological and four technical replicates. The growth curves of the wild type strain (ATCC 17978), LPS-deficient mutant (A-4-60, XH193), and LPS-modified mutant (C-1-12, XH198) in MH **(B)** and FBS **(C)**.

To investigate the effect of LPS on the serum resistance of *A. baumannii*, we measured the growth rate of the wild type strain (ATCC 17978), the LPS-deficient mutant (A-4-60, XH193), and the LPS-modified mutant (C-1-12, XH198) in FBS. There was significant difference in the growth rate of the wild type strain and LPS-deficient mutants in MH (**Figure 4A**). However, both the LPS-deficient mutant and LPS-modified mutant showed higher inhibition in FBS compared to that of the wild type strain. Both the LPS-deficient mutant and LPS-modified mutant exhibited a decrease in their final biomass (**Figures 4B,C**).

### Colistin-Resistant Mutation Alters the Antibiotic Resistance Profiles

To examine the effect of the LPS mutation on antibiotic resistance, we determined the MICs of several antibiotics (including quinolones, aminoglycosides, tetracycline, and β-lactams). **Table 1** shows the MICs of different antibiotics for *A. baumannii* ATCC 17978 and its colistin mutants. Overall, the changes in the MIC trends could be divided into two groups. The first group included seven strains that showed increased sensitivity to several types of antibiotics except colistin. All seven strains harbored mutations that disrupted the LPS biosynthesis pathway, leading to LPS loss. The second group contained only one strain: C-1-12 (XH198). This strain was resistant to all antibiotics except ampicillin and imipenem, and its colistin resistance mechanism involved an LPS modification. Thus, the LPS mutations resulted in differences in the antibiotic resistance profiles.

**Table 1:** Antimicrobial susceptibilities of *Acinetobacter baumannii* ATCC 17978 and its colistin mutants.

		Minimum inhibitory concentration (MIC; mg/L)^a^							
		
Strain	Lineage	CI	GM	AK	MC	TGC	AM	MP	IP
17978		0.25	0.75	2	0.75	0.38	48	0.38	0.38
XH191	A-1-60	0.125	0.38	0.5	<0.016	0.032	3	0.016	0.094
XH181	B-1-60	0.094	0.25	0.5	<0.016	0.047	2	0.012	0.125
XH192	A-2-60	0.25	1	0.5	0.016	<0.016	1	0.016	<0.002
XH182	B-2-60	0.125	0.094	0.19	0.032	0.064	1	0.008	0.064
XH183	B-3-60	0.094	0.38	0.5	0.032	0.032	3	0.016	0.125
XH193	A-4-60	0.094	0.5	1	0.016	0.094	3	0.032	0.125
XH184	B-4-60	0.125	0.19	0.5	0.032	0.064	1.5	0.008	0.064
XH198	C-1-12	1	2	8	1	3	12	0.5	0.25


*^a^CI, ciprofloxacin; GM, gentamicin; AK, amikacin; MC, minocycline; TGC, tigecycline; AM, ampicillin; MP, meropenem; IP, imipenem.*

## Discussion

In this study, we investigated the evolutionary pathway of *A. baumannii* that led to colistin resistance. Mutations in *pmrB. lpxC. lpxA*, and *lpxD* were detected early in the selection process. These results confirmed the findings of previous reports (Adams et al., 2009; Moffatt et al., 2010; Olaitan et al., 2014). In addition, four putative proteins, *A1S_1983. hepA. A1S_3026*, and *rsfS* were proposed to be involved in colistin resistance in this study. A1S_1983 is a putative exported protein. The ATP-dependent helicase HepA (A1S_2462) is a transcriptional regulator that activates transcription under stress conditions. The HepA mutation resulted in reduced exopolysaccharide coverage and increased retention of penicillin G compared to that of the wild type strain in *E. coli* strain; moreover, the mutant was more sensitive to norfloxacin, chloramphenicol, and gentamicin than the wild type (Lynch et al., 2007). A1S_3026 is a ribonuclease T2 family protein, and a mutation in this gene limits *A. baumannii*’s ability to colonize inanimate surfaces and bacterial mobility (Jacobs et al., 2014). RsfS (A1S_0570) is a ribosomal silencing factor that helps bacteria adapt to slow growth conditions (Hauser et al., 2012). The first protein is a membrane protein; its mutation would influence the integrity of the membrane, possibly leading to colistin resistance. The latter three proteins affected exopolysaccharide production, biofilm formation and bacterial adaptation, indicating new colistin resistance mechanisms. Recently, six genes (*vacJ. pldA. ttg2C. pheS*, and conserved hypothetical protein) were shown to have a role in colistin resistance (Thi Khanh Nhu et al., 2016). We also identified mutation in *vacJ*, but did not link the *vacJ* mutation to colistin resistance. This may be due to different mutations in *vacJ* in Nhu’s and our study. Moreover, these result also demonstrated the diversity of novel colistin resistance mechanism.

The LPS loss mutant showed a high fitness cost, which limited its spread in clinical environments. We identified two fitness compensatory mutations: A1S_3026 and RsfS in this study. These fitness compensatory mutations increase the fitness of the LPS loss mutant and promote its survival and spread in the clinical environment. However, we confirmed that the LPS-deficient mutants possessed an altered antibiotic resistance profile that was not observed in the LPS modification mutants. Colistin resistance in *A. baumannii* leads to changes in the antibiotic resistance profile (Moffatt et al., 2010; Lopez-Rojas et al., 2011b; Wand et al., 2015). The change in the antibiotic resistance profiles of the LPS-deficient mutants was caused by the increased permeability of the outer membrane to antibiotics (Moffatt et al., 2010). LPS in the outer membrane of *A. baumannii* acts as a highly selective permeability barrier (Nikaido, 2003). LPS loss removed the selective permeability of the barrier for antibiotics, whereas the LPS modification resulted in only minor changes in the permeability of the *A. baumannii* outer membrane. The *pmrB* mutant presented little or no change in susceptibility to these antibiotics (Wand et al., 2015). In this study, the *pmrB* mutant showed increased resistance to antibiotics. This may be due to changes in the permeability of the outer membrane by the *pmrB* mutation.

Previous studies have also demonstrated that the *pmrB* mutation resulted in no or a slight fitness cost (Durante-Mangoni et al., 2015; Wand et al., 2015). Our result confirmed these findings. These results explained why the LPS modification only resulted in a small change in the bacteria compared to the LPS loss. However, we did not determine the effect of colistin resistance-related mutations on the virulence of *A. baumannii* isolates *in vivo*. Colistin resistance was previously associated with lower *in vivo* fitness and reduced virulence in *A. baumannii* (Lopez-Rojas et al., 2011a).

In our study, LPS loss affected the growth rate of *A. baumannii* in nutrient-rich medium and serum. The loss of the *O*-ag capsule in *Salmonella typhimurium* was also reported to reduce the resistance to serum but did not affect the growth rate (Marshall and Gunn, 2015). The LPS loss was not shown to contribute to serum resistance in *A. baumannii* (Moffatt et al., 2013). However, our results did not support that conclusion. LPS modification decreased the growth rate of *A. baumannii* in serum and in nutrient-rich medium. The LPS modified mutation also contributed to serum resistance in *A. baumannii*. The conflict between Moffatt and our results may be caused by the different genetic backgrounds of ATCC 19606 used in Moffatt’s study and ATCC 17978 used in our study. Both of the LPS mutants showed a decreased the final biomass, whereas serum resistance was normal following incubation in serum for 30 min (Moffatt et al., 2013). The decreased biomass indicated that the serum resistance assay required further improvements.

Overall, colistin resistance *A. baumannii* rapidly developed. We not only confirmed the existence of a previously reported colistin resistance mechanism, but also identified four novel putative proteins involved in colistin resistance. The compensatory mutations partially or completely recovered the LPS-deficient mutant’s fitness, suggesting that compensatory mutations played an important role in the emergence and spread of colistin resistant *A. baumannii*.

### Nucleotide Sequence Accession Numbers

The whole genome shotgun sequencing results for B-1-60 (XH181), B-2-60(XH182), B-3-15(XH183), B-4-60(XH184), A-1 -60(XH191), A-2-60(XH192), A-4-60(XH193), and C-1-12(XH198) have been deposited at DDBJ/EMBL/GenBank under the accession numbers MDWH00000000, MDWJ00000000, MDWK00000000, MDWL00000000, MDWG00000000, MDWI00000000, MDWF00000000, and MDWM00000000, respectively.

## Author Contributions

XH and YY conceived and designed the study. XM, NW, XL, and KS performed the experiments. XH, ZZ, and YY performed data analysis and drafted the manuscript. All authors reviewed and approved the final manuscript.

## Conflict of Interest Statement

The authors declare that the research was conducted in the absence of any commercial or financial relationships that could be construed as a potential conflict of interest.

The reviewer YS and handling Editor declared their shared affiliation and the handling Editor states that the process nevertheless met the standards of a fair and objective review.

## References

[B1] Adams M. D. Nickel G. C. Bajaksouzian S. Lavender H. Murthy A. R. Jacobs M. R. (2009). Resistance to colistin in *Acinetobacter baumannii* associated with mutations in the PmrAB two-component system. *Antimicrob. Agents Chemother.* 53 3628–3634. 10.1128/AAC.00284-09 19528270 PMC2737849

[B2] Andersson D. I. Hughes D. (2010). Antibiotic resistance and its cost: is it possible to reverse resistance? *Nat. Rev. Microbiol.* 8 260–271. 10.1038/nrmicro2319 20208551

[B3] Baker S. Duy P. T. Nga T. V. Dung T. T. Phat V. V. Chau T. T. (2013). Fitness benefits in fluoroquinolone-resistant *Salmonella* Typhi in the absence of antimicrobial pressure. *Elife* 2:e01229 10.7554/eLife.01229 PMC3857714 24327559

[B4] Beceiro A. Llobet E. Aranda J. Bengoechea J. A. Doumith M. Hornsey M. (2011). Phosphoethanolamine modification of lipid A in colistin-resistant variants of *Acinetobacter baumannii* mediated by the pmrAB two-component regulatory system. *Antimicrob. Agents Chemother.* 55 3370–3379. 10.1128/AAC.00079-11 21576434 PMC3122444

[B5] Beceiro A. Moreno A. Fernandez N. Vallejo J. A. Aranda J. Adler B. (2014). Biological cost of different mechanisms of colistin resistance and their impact on virulence in *Acinetobacter baumannii*. *Antimicrob. Agents Chemother.* 58 518–526. 10.1128/AAC.01597-13 24189257 PMC3910726

[B6] Clinical and Laboratory Standards Institute (2014). *Performance Standards for Antimicrobial Susceptibility Testing: 24th Informational Supplement. CLSI Document M100-S24*. Wayne, PA: Clinical and Laboratory Standards Institute.

[B7] Deatherage D. E. Barrick J. E. (2014). Identification of mutations in laboratory-evolved microbes from next-generation sequencing data using breseq. *Methods Mol. Biol.* 1151 165–188. 10.1007/978-1-4939-0554-6_12 24838886 PMC4239701

[B8] Durante-Mangoni E. Del Franco M. Andini R. Bernardo M. Giannouli M. Zarrilli R. (2015). Emergence of colistin resistance without loss of fitness and virulence after prolonged colistin administration in a patient with extensively drug-resistant *Acinetobacter baumannii*. *Diagn. Microbiol. Infect. Dis.* 82 222–226. 10.1016/j.diagmicrobio.2015.03.013 25858028

[B9] Durante-Mangoni E. Utili R. Zarrilli R. (2014). Combination therapy in severe *Acinetobacter baumannii* infections: an update on the evidence to date. *Future Microbiol.* 9 773–789. 10.2217/fmb.14.34 25046524

[B10] European Committee on Antimicrobial Susceptibility Testing (2015). *Breakpoint Tables for Interpretation of MICs and Zone Diameters. Version 2.0. EUCAST; 2012.* Basel: European Committee on Antimicrobial Susceptibility Testing.

[B11] Falagas M. E. Bliziotis I. A. Kasiakou S. K. Samonis G. Athanassopoulou P. Michalopoulos A. (2005). Outcome of infections due to pandrug-resistant (PDR) Gram-negative bacteria. *BMC Infect. Dis.* 5:24 10.1186/1471-2334-5-24 PMC1087841 15819983

[B12] Giske C. G. Monnet D. L. Cars O. Carmeli Y. ReAct-Action on Antibiotic Resistance. (2008). Clinical and economic impact of common multidrug-resistant gram-negative bacilli. *Antimicrob. Agents Chemother.* 52 813–821. 10.1128/AAC.01169-07 18070961 PMC2258516

[B13] Hauser R. Pech M. Kijek J. Yamamoto H. Titz B. Naeve F. (2012). RsfA (YbeB) proteins are conserved ribosomal silencing factors. *PLoS Genet.* 8:e1002815 10.1371/journal.pgen.1002815 PMC3400551 22829778

[B14] Hua X. Zhou H. Jiang Y. Feng Y. Chen Q. Ruan Z. (2012). Genome sequences of two multidrug-resistant *Acinetobacter baumannii* strains isolated from a patient before and after treatment with tigecycline. *J. Bacteriol.* 194 6979–6980. 10.1128/JB.01887-12 23209232 PMC3510637

[B15] Jacobs A. C. Blanchard C. E. Catherman S. C. Dunman P. M. Murata Y. (2014). An ribonuclease T2 family protein modulates *Acinetobacter baumannii* abiotic surface colonization. *PLoS ONE* 9:e85729 10.1371/journal.pone.0085729 PMC3904860 24489668

[B16] Li X. Liu L. Ji J. Chen Q. Hua X. Jiang Y. (2015). Tigecycline resistance in *Acinetobacter baumannii* mediated by frameshift mutation in plsC, encoding 1-acyl-sn-glycerol-3-phosphate acyltransferase. *Eur. J. Clin. Microbiol. Infect. Dis.* 34 625–631. 10.1007/s10096-014-2272-y 25407371

[B17] Lopez-Rojas R. Dominguez-Herrera J. McConnell M. J. Docobo-Perez F. Smani Y. Fernandez-Reyes M. (2011a). Impaired virulence and in vivo fitness of colistin-resistant *Acinetobacter baumannii*. *J. Infect. Dis.* 203 545–548. 10.1093/infdis/jiq086 21216865 PMC3071218

[B18] Lopez-Rojas R. Jimenez-Mejias M. E. Lepe J. A. Pachon J. (2011b). *Acinetobacter baumannii* resistant to colistin alters its antibiotic resistance profile: a case report from Spain. *J. Infect. Dis.* 204 1147–1148. 10.1093/infdis/jir476 21881133

[B19] Lynch S. V. Dixon L. Benoit M. R. Brodie E. L. Keyhan M. Hu P. (2007). Role of the rapA gene in controlling antibiotic resistance of *Escherichia coli* biofilms. *Antimicrob. Agents Chemother.* 51 3650–3658. 10.1128/AAC.00601-07 17664315 PMC2043260

[B20] Magiorakos A. P. Srinivasan A. Carey R. B. Carmeli Y. Falagas M. E. Giske C. G. (2012). Multidrug-resistant, extensively drug-resistant and pandrug-resistant bacteria: an international expert proposal for interim standard definitions for acquired resistance. *Clin. Microbiol. Infect.* 18 268–281. 10.1111/j.1469-0691.2011.03570.x 21793988

[B21] Marcusson L. L. Frimodt-Moller N. Hughes D. (2009). Interplay in the selection of fluoroquinolone resistance and bacterial fitness. *PLoS Pathog.* 5:e1000541 10.1371/journal.ppat.1000541 PMC2714960 19662169

[B22] Marshall J. M. Gunn J. S. (2015). The O-antigen capsule of *Salmonella enterica* Serovar Typhimurium facilitates serum resistance and surface expression of FliC. *Infect. Immun.* 83 3946–3959. 10.1128/IAI.00634-15 26195553 PMC4567616

[B23] Moffatt J. H. Harper M. Harrison P. Hale J. D. Vinogradov E. Seemann T. (2010). Colistin resistance in *Acinetobacter baumannii* is mediated by complete loss of lipopolysaccharide production. *Antimicrob. Agents Chemother.* 54 4971–4977. 10.1128/AAC.00834-10 20855724 PMC2981238

[B24] Moffatt J. H. Harper M. Mansell A. Crane B. Fitzsimons T. C. Nation R. L. (2013). Lipopolysaccharide-deficient *Acinetobacter baumannii* shows altered signaling through host Toll-like receptors and increased susceptibility to the host antimicrobial peptide LL-37. *Infect. Immun.* 81 684–689. 10.1128/iai.01362-12 23250952 PMC3584870

[B25] Nikaido H. (2003). Molecular basis of bacterial outer membrane permeability revisited. *Microbiol. Mol. Biol. Rev.* 67 593–656. 10.1128/MMBR.67.4.593-656.2003 14665678 PMC309051

[B26] Olaitan A. O. Morand S. Rolain J. M. (2014). Mechanisms of polymyxin resistance: acquired and intrinsic resistance in bacteria. *Front. Microbiol.* 5:643 10.3389/fmicb.2014.00643 PMC4244539 25505462

[B27] Peleg A. Y. Seifert H. Paterson D. L. (2008). *Acinetobacter baumannii*: emergence of a successful pathogen. *Clin. Microbiol. Rev.* 21 538–582. 10.1128/CMR.00058-07 18625687 PMC2493088

[B28] R Development Core Team (2015). *R: A Language and Environment for Statistical Computing*. Vienna: R Foundation for Statistical Computing.

[B29] Thi Khanh Nhu N. Riordan D. W. Do Hoang Nhu T. Thanh D. P. Thwaites G. (2016). The induction and identification of novel Colistin resistance mutations in *Acinetobacter baumannii* and their implications. *Sci Rep.* 6:28291 10.1038/srep28291 PMC4916428 27329501

[B30] Trindade S. Sousa A. Xavier K. B. Dionisio F. Ferreira M. G. Gordo I. (2009). Positive epistasis drives the acquisition of multidrug resistance. *PLoS Genet.* 5:e1000578 10.1371/journal.pgen.1000578 PMC2706973 19629166

[B31] Wand M. E. Bock L. J. Bonney L. C. Sutton J. M. (2015). Retention of virulence following adaptation to colistin in *Acinetobacter baumannii* reflects the mechanism of resistance. *J. Antimicrob. Chemother.* 70 2209–2216. 10.1093/jac/dkv097 25904728

