# Supplementary material for: The Effect of Colistin Resistance-Associated Mutations on the Fitness of Acinetobacter baumannii
Source: Front Microbiol. 2016 Nov 1;7:1715. doi: 10.3389/fmicb.2016.01715 (PMC5088200; doi:10.3389/fmicb.2016.01715)
Supplement: Supplementary file 1 [file Table_1.DOCX]

Table S1: Characterization of laboratory-evolved colistin resistant *A. baumannii* strains

| Strain | Lineage | Putative mutation(s) causing reduced susceptibility to COL or fitness change | COL MIC (mg/L) | Relative growth rate |
| --- | --- | --- | --- | --- |
| ATCC 17978 | 17978 | NA | 0.19 | 1.00±0.13 |
| XH194 | A-1-15 | lpxC::ISAba1 | 64 | 0.75±0.09 |
| XH185 | A-1-30 | lpxC::ISAba1 | 48 | 0.72±0.03 |
| XH188 | A-1-45 | lpxC::ISAba1, A1S_1983 A52G | 256 | 0.55±0.04 |
| XH191 | A-1-60 | lpxC::ISAba1, A1S_1983 A52G, rpoC R95C | 256 | 0.57±0.05 |
| XH195 | A-2-15 | lpxD::ISAba1 | 32 | 0.30±0.02 |
| XH186 | A-2-30 | lpxD::ISAba1 | 32 | 0.40±0.02 |
| XH189 | A-2-45 | lpxD::ISAba1, hepA Q590* | 96 | 0.33±0.03 |
| XH192 | A-2-60 | lpxD::ISAba1, hepA Q590*, A1S_3026 Q119*, adeS S57F | >256 | 0.53±0.01 |
| XH196 | A-3-15 | lpxA I76K | 24 | 0.74±0.03 |
| XH197 | A-4-15 | lpxA I76K | 96 | 0.67±0.13 |
| XH187 | A-4-30 | lpxA I76K, A1S_0570 L112P (36%) | 128 | 0.76±0.13 |
| XH190 | A-4-45 | lpxA I76K, A1S_0570 L112P, Dup 100 kb | >256 | 1.11±0.07 |
| XH193 | A-4-60 | lpxA I76K, A1S_0570 L112P, Dup 100 kb | >256 | 0.75±0.04 |
| XH181 | B-1-60 | lpxC::ISAba1 | 48 | 0.78±0.10 |
| XH182 | B-2-60 | lpxD::ISAba1 | 32 | 0.34±0.06 |
| XH183 | B-3-15 | lpxA I76K | 64 | 0.78±0.04 |
| XH184 | B-4-60 | lpxA I76K | 192 | 0.91±0.15 |
| XH198 | C-1-12 | pmrB G272D | 6 | 0.91±0.04 |

COL, colistin; NA, not applicable; dup, duplication
