# Supplementary material for: The Effect of Colistin Resistance-Associated Mutations on the Fitness of Acinetobacter baumannii
Source: Front Microbiol. 2016 Nov 1;7:1715. doi: 10.3389/fmicb.2016.01715 (PMC5088200; doi:10.3389/fmicb.2016.01715)
Supplement: Supplementary file 2 [file Table_2.DOCX]

Table S2: The primers used for RT-PCR and mutation verification

| primer | sequence |
| --- | --- |
| RT-PCR^a^  dup100_R1 | TTATCAACCACGCTTACCC |
| dup100_F1 | AATCACCTCACCCTCAAC |
| dup100up_F1 | TTGCTCACCAGTGTTTTC |
| dup100up_R1 | GTGAGTGGGTAATAAAGAAG |
| dup100down_F1 | CAACATAACCCACTCCTC |
| dup100down_R1 | TACATTCTCCATACCCGC |
| Verification |  |
| pmrB_BF1 | GGATCCAAACGACTGATTTGGGGCACCT |
| pmrB_SR1 | GTCGACTTCCTAAGCCATAACTTATGGA |
| lpxA_BF1 | GGATCCCACACCAAAAAACAGAGCAGGC |
| lpxA_SR1 | GTCGACAGCAAATCAATACAAGAACAAG |
| 1983_2F1 | ATCATGCCGTCGAATACT |
| 1983_2R1 | TGGTTGTATGTGGTGGTG |
| 1754_2F1 | TGTTATCTTTTGCGGCTGT |
| 1754_2R1 | TATTGGGAGTTGTGGTGG |
| 2462_2F1 | GTGGCGCTTTTTTATTACTC |
| 2462_2R1 | AGAAAATGGCGAGCAAGAA |
| 3026_2F1 | GTGGCAGATGCATGTAAA |
| 3026_2R1 | GGCAGCGTTAAGTGAAAT |
| 0570_2F1 | TGAGGGTAAAGGTGGGTG |
| 0570_2R1 | CGGAGTTTGTGTATGGGT |
| 0288_2F1 | TCCGTATTGGTCTTGCGT |
| 0288_2R1 | CCATTTTCTTCGCAGCTTT |
| lpxD_F1 | CTGCTCTGTTTTTTGGTGT |
| lpxD_R1 | CTGGTTATGGGGTAATTGATCT |
| lpxC_F1 | CAACAAACCGGAAAACACA |
| lpxC_R1 | CAGAGCCAAGAAAGCGTAA |

^a^To detect the existence of the duplication of XH193, we design three primers to perform duplication verification: up: the upstream of duplication fragment; down: the downstream of duplication fragment; center: within the duplication fragment.
