# Supplementary material for: The Effect of Colistin Resistance-Associated Mutations on the Fitness of Acinetobacter baumannii
Source: Front Microbiol. 2016 Nov 1;7:1715. doi: 10.3389/fmicb.2016.01715 (PMC5088200; doi:10.3389/fmicb.2016.01715)
Supplement: Supplementary file 3 [file Table_3.DOCX]

Table S3: Summary of colistin resistance and fitness costs of the mutations in *A. baumannii*

| Mutation | Resistance | Fitness | Gene | Description | Mutation compensatory |
| --- | --- | --- | --- | --- | --- |
| lpxC | + | ↓ | lpxC | UDP-3-O-acyl N-acetylglycosamine deacetylase |  |
| lpxD | + | ↓ | lpxD | UDP-3-O-[3-hydroxymyristoyl] glucosamine N-acyltransferase |  |
| lpxA | + | ↓ | lpxA | UDP-N-acetylglucosamine O-acyltransferase |  |
| pmrB | + | ↓ | pmrB | Sensor protein BasS/PmrB |  |
| A1S_1983 | + | ↓ |  | hypothetical membrance protein |  |
| A1S_2462 | + | - | hepA | Polypeptide: RNA Polymerase (RNAP)-binding ATPase and RNAP recycling factor |  |
| A1S_3026 | + | **↑** |  | ribonuclease T2 family protein | lpxD, hepA(XH189) |
| A1S_0570 | + | ↑ |  | hypothetical protein (RsfS) | lpxA(XH197) |
