# Supplementary material for: The Effect of Colistin Resistance-Associated Mutations on the Fitness of Acinetobacter baumannii
Source: Front Microbiol. 2016 Nov 1;7:1715. doi: 10.3389/fmicb.2016.01715 (PMC5088200; doi:10.3389/fmicb.2016.01715)
Supplement: Supplementary file 4 [file Table_4.DOCX]

Table S4: the mutation detected in laboratory-evolved colistin resistant *A. baumannii*

| Position | DNA change | Amino acid substitution | Gene description | Locus_tag | Gene |
| --- | --- | --- | --- | --- | --- |
|  |  |  |  |  |  |
| **B-1-60(XH181)** |  |  |  |  |  |
| 673543 | AAC=>ATC | V93D | lipoprotein precursor (VacJ) transmembrane | A1S_0622 | VacJ |
| 2526939 | CGT=>GGT | R303G | phosphoenolpyruvate synthase | A1S_2164 |  |
|  |  | lpxC::IS*Aba1* |  |  |  |
| **B-2-60(XH182)** |  |  |  |  |  |
| 2467093 | CAA=>CCA | L55W | tRNA delta(2)-isopentenylpyrophosphate transferase | A1S_2113 | miaA |
|  |  | lpxD::IS*Aba1* |  |  |  |
| **B-3-15(XH183)** |  |  |  |  |  |
| 2277608 | TAT=>TTT | I76K | UDP-N-acetylglucosamine acyltransferase | A1S_1965 | lpxA |
| 2488890 | ATC=>TTC | D346E | outer membrane protein | A1S_2132 |  |
| 2695714 | ATT=>GTT | I309V | hypothetical protein | A1S_2327 |  |
| 3108496 | TGT=>TGA | T41S | cell division protein | A1S_2682 | ftsJ |
| 3244671 | GCA=>GAA | A230E | hypothetical protein | A1S_2798 |  |
|  |  |  |  |  |  |
| **B-4-60(XH184)** |  |  |  |  |  |
| 2277608 | TAT=>TTT | I76K | UDP-N-acetylglucosamine acyltransferase | A1S_1965 | lpxA |
| 2488890 | ATC=>TTC | D346E | outer membrane protein | A1S_2132 |  |
| 3108496 | TGT=>TGA | T41S | cell division protein | A1S_2682 | ftsJ |
| 3244671 | GCA=>GAA | A230E | hypothetical protein | A1S_2798 |  |
|  |  |  |  |  |  |
| **A-1-60(XH191)** |  |  |  |  |  |
| 310173 | CGT=>TGT | R95C | DNA-directed RNA polymerase subunit beta' | A1S_0288 | rpoC |
| 673543 | AAC=>ATC | V93D | lipoprotein precursor (VacJ) transmembrane | A1S_0622 | VacJ |
| 2295887 | GCC=>GGC | A52G | signal peptide | A1S_1983 |  |
|  |  | lpxC::IS*Aba1* |  |  |  |
| **A-2-60(XH192)** |  |  |  |  |  |
| 2042072 | TCC=>TTC | S57F | AdeS | A1S_1754 | AdeS |
| 2467093 | CAA=>CCA | L55W | tRNA delta(2)-isopentenylpyrophosphate transferase | A1S_2113 | miaA |
| 2851575 | TTG=>TTA | Q590* | ATP-dependent helicase HepA | A1S_2462 | HepA |
| 3512095 | TTG=>TTA | Q119* | hypothetical protein | A1S_3026 |  |
| 3580330 | ACG=>AC- | R53X | toluene tolerance efflux transporter | A1S_3101 |  |
|  |  | lpxD::IS*Aba1* |  |  |  |
| **A-4-60(XH193)** |  |  |  |  |  |
| 618745 | TAG=>TGG | L112P | hypothetical protein | A1S_0570 |  |
| 2277608 | TAT=>TTT | I76K | UDP-N-acetylglucosamine acyltransferase | A1S_1965 | lpxA |
| 2488890 | ATC=>TTC | D346E | outer membrane protein | A1S_2132 |  |
| 3108496 | TGT=>TGA | T41S | cell division protein | A1S_2682 | ftsJ |
| 3244671 | GCA=>GAA | A230E | hypothetical protein | A1S_2798 |  |
|  |  |  |  |  |  |
| **C-1-12(XH198)** |  |  |  |  |  |
| 1799260 | AAA=>AAC | F568V | organic solvent tolerance protein | A1S_1546 | ostA |
| 1836841 | GGG=>CGG | G387R | replicative DNA helicase | A1S_1585 | dnaB |
| 1904060 | TAA=>TTA | *287L | peptidyl-prolyl cis-trans isomerase | A1S_1638 | PPIase |
| 2042143 | GGT=>AGT | G81S | AdeS | A1S_1754 | adeS |
| 2286491 | CAT=>CGT | M50T | undecaprenyl pyrophosphate synthetase | A1S_1973 | ups |
| 2326517 | TAT=>AAT | Y21N | biotin carboxylase (A subunit of acetyl-CoA carboxylase) | A1S_2011 | acc |
| 2896159 | ATT=>AAT | N173I | GTP-binding protein (Obg) | A1S_2498 | obg |
| 3190295 | ACC=>ATC | G272D | two-component sensor kinase transcription regulator protein | A1S_2750 | pmrB |
| 3794200 | TGC=>TGT | A360T | acetate permease | A1S_3300 | actP |
